# Safety and Outcomes of Alcohol Septal Ablation Prior to Transcatheter Mitral Valve Replacement

**DOI:** 10.1016/j.jscai.2022.100396

**Published:** 2022-07-02

**Authors:** Mohamed Elhadi, Mayra Guerrero, Jeremy D. Collins, Charanjit S. Rihal, Mackram F. Eleid

**Affiliations:** Department of Cardiovascular Medicine, Mayo Clinic, Rochester, Minnesota

**Keywords:** catheter-based coronary and valvular interventions, septal ablation, valvular heart disease

## Abstract

**Background:**

Patients undergoing transcatheter mitral valve replacement (TMVR) for mitral valve disease caused by severe mitral annular calcification are at risk of left ventricular outflow obstruction. Preemptive alcohol septal ablation (ASA) can potentially mitigate the risk of this complication and is well established in patients with hypertrophic obstructive cardiomyopathy (HCM).

**Methods:**

This retrospective study compared procedural characteristics and outcomes in patients who underwent ASA for TMVR vs HCM.

**Results:**

In total, 102 patients were included, 22 in the TMVR group and 80 in the HCM group. Echocardiography demonstrated increased septal wall thickness in the HCM group (19 ​± ​3.1 ​mm vs 12.7 ​± ​2.0 ​mm; *P* ​< ​.001). The mean volume of ethanol injected was higher in the HCM group (1.4 ​± ​0.49 ​mL vs 0.8 ​± ​0.2 ​mL; *P* ​< ​*.*001). The average neo–left ventricular outflow tract area increased significantly after ASA in the patients undergoing TMVR (135 ​± ​89 ​mm^2^ vs 233 ​± ​111 ​mm^2^; *P* ​< ​.001). Six patients in the TMVR group did not achieve an adequate increase in the neo–left ventricular outflow tract area and required further procedures after ASA. The incidence of post-ASA complete heart block requiring a permanent pacemaker tended to be higher in the TMVR group (35% vs 21%; *P* ​= ​*.*195). No patients in either group had ventricular arrhythmia or stroke. Major bleeding complications were 4% in the HCM group and 0 in the TMVR group. The 30-day mortality was 4% in the HCM group and 0 in the TMVR group; however, 1 patient died at 37 ​days in the TMVR group, presumably from late heart block.

**Conclusions:**

Preemptive ASA in patients undergoing TMVR demonstrated safety and short-term clinical outcomes similar to patients with HCM.

## Highlights

• Left ventricular outflow obstruction is a transcatheter mitral valve complication.
• Alcohol septal ablation can mitigate the risk of this lethal complication.
• Septal ablation before transcatheter mitral valve replacement is safe and feasible.
• Outcomes were similar to those of obstructive hypertrophic cardiomyopathy.

## Central Illustration

**Figure:**
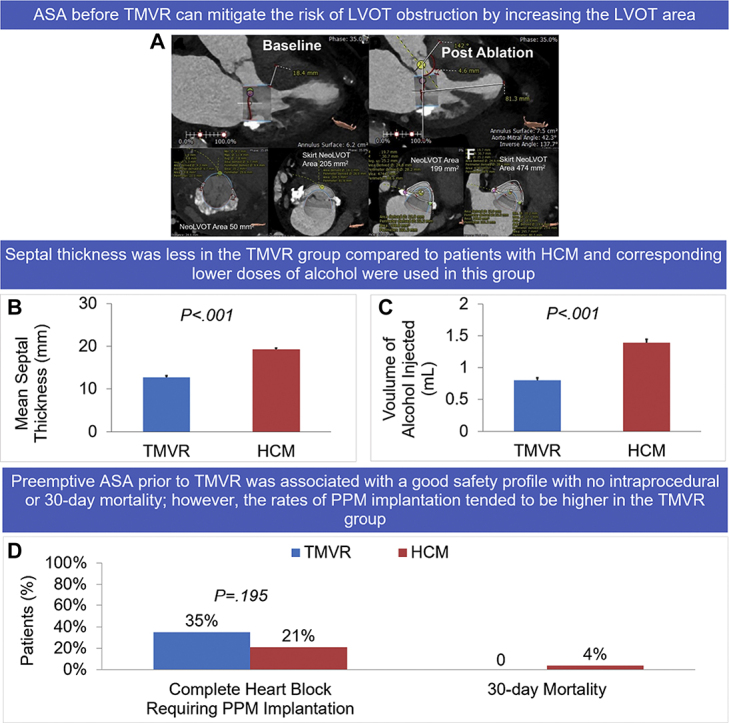

## Introduction

Mitral valve disease is the most common valvular heart disorder in the elderly population, with a prevalence of 10% in patients aged >75 ​years.^1^ Transcatheter mitral valve replacement (TMVR) is an emerging and developing therapy for severe symptomatic mitral valve disease. Patients who are poor surgical candidates with severe mitral valve disease represent a group that stands to potentially benefit from this transcatheter and minimally invasive procedure. Among this cohort, patients with mitral annular calcification (MAC) are often at risk of left ventricular outflow tract (LVOT) obstruction after deployment of the valve because of anterior mitral leaflet displacement. Data from 2 multicenter registries, the Transcatheter Valve Therapies Registry and TMVR in MAC global registry, demonstrated a 10% and 11.2% rate of LVOT obstruction with TMVR, respectively.^2^^,^^3^ Urgent alcohol septal ablation (ASA) was used initially as a bail-out strategy in patients who developed this potentially fatal complication after valve deployment.^4^ Preemptive ASA before TMVR is a current strategy that has the potential to prevent LVOT obstruction after TMVR by increasing the LVOT area.^5^ Although the safety and efficacy of ASA for hypertrophic obstructive cardiomyopathy (HCM) with symptomatic dynamic left ventricular outflow obstruction are established, its role in severe MAC being considered for TMVR is unclear. Therefore, the objective of this study was to assess the safety of preemptive ASA in patients undergoing TMVR by comparing outcomes with those of patients undergoing ASA for HCM.

## Materials and methods

### Study design and participants

This is a single-center retrospective observational cohort study comparing safety and outcomes in 2 groups of patients treated at a tertiary referral center in Rochester, Minnesota. The TMVR group consisted of all patients who underwent preemptive ASA preparation for TMVR. The HCM group consisted of consecutive patients who underwent ASA for HCM with symptomatic dynamic LVOT obstruction. Patients in both the groups underwent ASA during the same time period, from May 2016 to August 2021. The study was approved by the Mayo Clinic Institutional Review Board, allowing retrospective review of medical records and granting a waiver of informed consent.

#### HCM group

The HCM group consisted of patients who underwent ASA for severe symptoms of dyspnea (New York Heart Association class III and IV), angina, or syncope. The criteria also included the following: (1) findings of severe dynamic LVOT obstruction with systolic anterior motion of the mitral valve (defined as an LVOT gradient of >50 ​mm Hg at rest or with provocation), (2) suitable coronary anatomy, (3) a diastolic septal thickness of ≥15 ​mm, (4) the absence of primary mitral valve disease, and (5) no indication for concomitant surgical intervention. At our institution, the choice of surgical myectomy or ASA was made through a shared decision-making process after a discussion of the risks and benefits of each alternative. Younger patients and those with specific anatomic considerations favoring surgical myectomy most often underwent surgery, whereas older patients and those with a larger burden of comorbidities more often were recommended to undergo ASA.

#### TMVR group

**Figure. 1:**
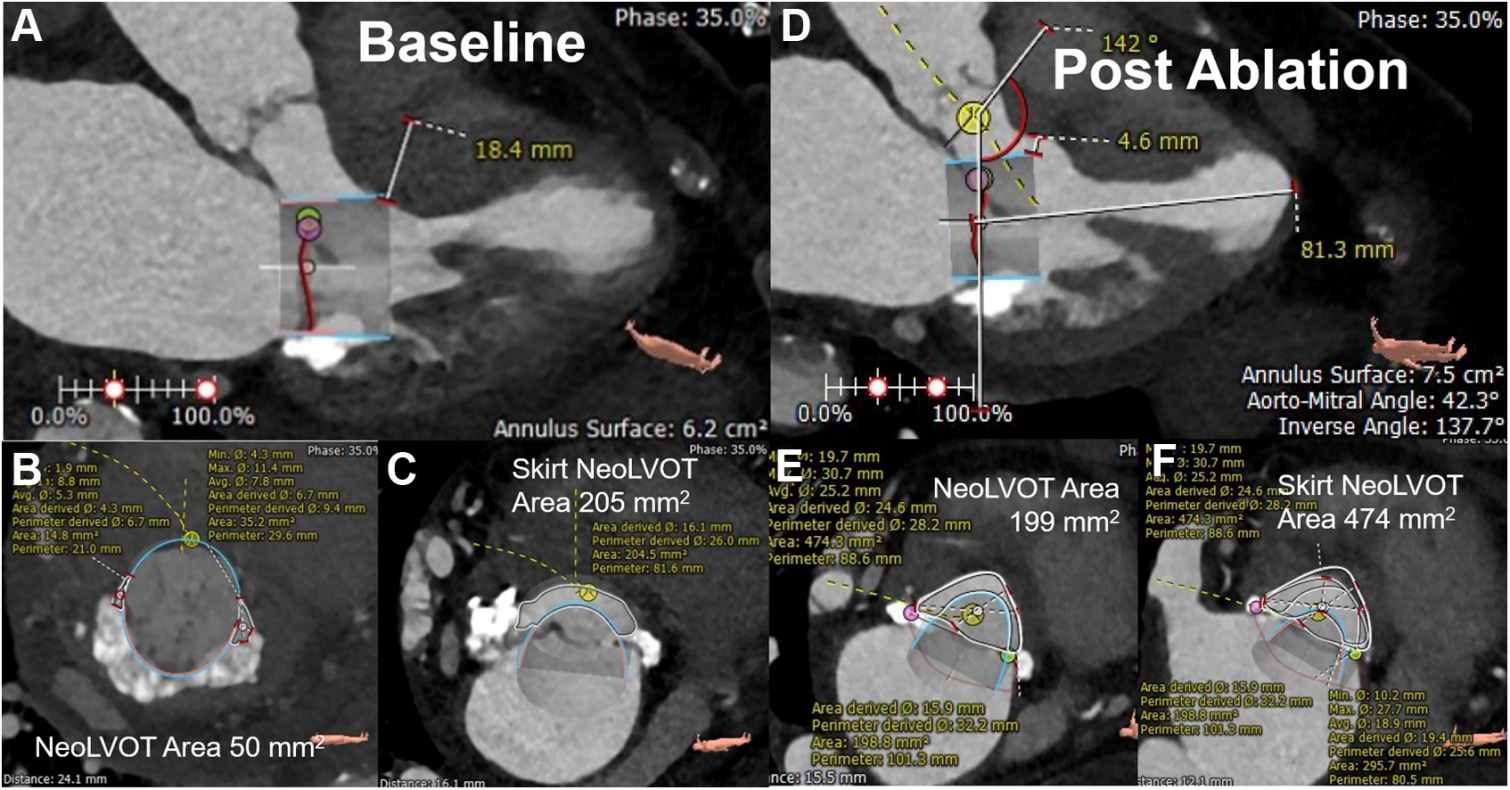
**Baseline and post-ASA frame neo-LVOT and skirt neo-LVOT areas.** Cardiac computed tomography images with a virtual valve in the mitral position demonstrating a change in the LVOT anatomy after ASA (**A-F**). Pre-ASA frame neo-LVOT area increased from 50 ​mm^2^ to 199 ​mm^2^ after ASA. Pre-ASA skirt neo-LVOT area increased from 205 ​mm^2^ to 474 ​mm^2^ after ASA. ASA, alcohol septal ablation; LVOT, left ventricular outflow tract.

The patients in the TMVR group had severe symptomatic mitral valve stenosis and/or regurgitation with severe MAC. Therefore, these patients were deemed as high-risk candidates for traditional mitral valve surgery by a cardiac surgeon. In addition, these patients had an LVOT obstruction risk assessment that was facilitated by cardiac-gated computed tomography (CT) angiography. TMVR implantation was simulated using a virtual valve with a size that was same as that of the proposed valve. The cross-sectional area between the most ventricular edge of the simulated valve and the basal anteroseptal wall of the left ventricle was measured as the neo-LVOT, representing an estimate of the true LVOT area after deployment of the valve (Figure 1). Patients with a neo-LVOT area of ≤189 ​mm^2^ were the patients at risk of LVOT obstruction^6^ who underwent preemptive ASA.

**Central Illustration:**
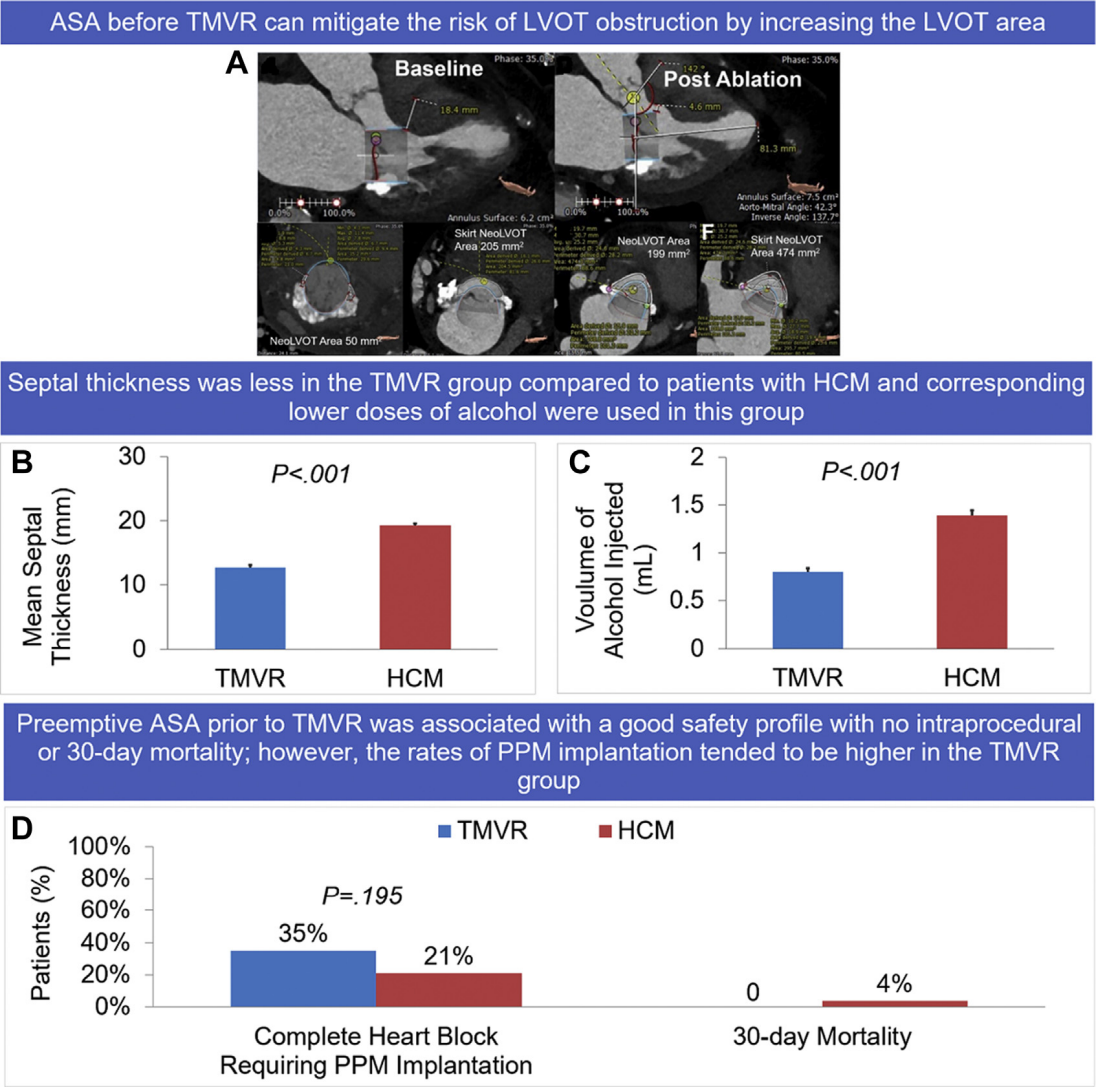
**Safety of alcohol septal ablation for septal reduction prior to transcatheter mitral valve replacement compared to obstructivehypertrophic obstructive cardiomyopathy.** (**A**) Cardiac computed tomography images with virtual valve in the mitral position demonstrating a change in the LVOT anatomy after ASA. (**B**) The mean septal thickness (in millimeters) was measured on pre-ASA echocardiography and was higher in the HCM group (19.3 ​± ​3.1 ​mm) than in the TMVR group (12.7 ​± ​2 ​mm) ​(*P* ​< ​.001). (**C**) The mean volume of alcohol injected during the ASA procedure was also higher in the HCM group (1.4 ​± ​0.49 ​mL) than in the TMVR group (0.8 ​± ​0.2 ​mL) ​(*P* ​< ​.001). (**D**) The incidence of complete heart block requiring PPM after ASA was 35% in the TMVR group and 21% in the HCM group (*P* ​= ​.195). The 30-day mortality was 0 in the TMVR group and 3.8% in the HCM group. ASA, alcohol septal ablation; HCM, hypertrophic obstructive cardiomyopathy; LVOT, left ventricular outflow tract; PPM, permanent pacemaker; TMVR, transcatheter mitral valve replacement.

### ASA

ASA was performed using standard and previously described techniques.^7^ First, a temporary transvenous pacemaker was implanted in patients without a preexisting pacemaker. Next, left coronary angiography was performed to identify the septal perforator arteries branching off the left anterior descending artery. An over-the-wire angioplasty balloon was advanced into the target septal perforator artery. The balloon was then inflated, causing occlusion of the septal perforator artery. The contrast was then injected to ensure complete sealing of the target septal perforator artery with no spill-back into the left anterior descending or collateral flow. Next, echocardiographic microbubble contrast was injected into the septal perforator artery. Transthoracic echocardiography then confirmed that the injected septal artery supplied the target area of the septum. According to the maximal septal thickness, 0.5 to 3 ​mL of 98% dehydrated ethanol was injected into the septal perforator artery, causing a localized infarction in the basal septum. In patients with HCM, simultaneous left ventricular and aortic pressures were measured before and after ablation to confirm the relief of dynamic obstruction. In patients with planned TMVRs, LVOT gradients were not present at baseline and, thus, were not assessed during ASA. Patients were admitted after the ASA procedure for at least 48 ​hours to monitor for arrhythmias.

### Variables

Baseline characteristics were collected retrospectively from the medical record for both groups. Pre-ASA echocardiographic data were collected, including left ventricular ejection fraction, interventricular septal thickness, posterior wall thickness, and left ventricular mass index. For the TMVR group, data were collected regarding the type of mitral valve disease (ie, mitral stenosis or mitral regurgitation or mixed), severity, valve area, gradient, and concomitant valve disease. Procedural characteristics of ASA, including the number of septal perforators injected and the volume of ethanol, were collected. For the TMVR group, we also collected details of LVOT, frame neo-LVOT, and skirt neo-LVOT areas analyzed from cardiac CT before and after ASA using 3mensio software (Pie Medical Imaging BV) by an interventional cardiologist (M.G. or M.F.E). Data were also collected on patients who required further procedures after ASA to improve the neo-LVOT. These procedures were radiofrequency ablation of the septum and laceration of the anterior mitral leaflet to prevent outflow obstruction (LAMPOON). The primary outcome variables consisted of the complications of ASA, in-hospital mortality, 30-day mortality, and survival at 1 ​year. Relevant complications included complete heart block (CHB), major bleeding, stroke, and sustained ventricular tachycardia. Major bleeding was defined using the Bleeding Academic Research Consortium criteria and included all instances of Bleeding Academic Research Consortium types 3a to 3c.^8^ All data were collected from the electronic medical record, and follow-up was assessed at the latest clinical interaction or via a phone call.

### Statistical analysis

Continuous data were presented as mean ​± ​standard deviation and categorical data were presented as n (%). χ^2^ analysis was conducted for categorical variables, and 2-sample unpaired *t* test was conducted for continuous variables. Hypothesis testing was 2-tailed, and we tested for any statistically significant differences between the 2 groups for the baseline and outcome variables. Survival was estimated using the Kaplan-Meier method with the log-rank test. A *P* value of <.05 was considered significant. All statistical analyses were performed using IBM SPSS Statistics for Windows, version 28.0 (IBM Corp).

## Results

### Baseline characteristics

**Figure. 2:**
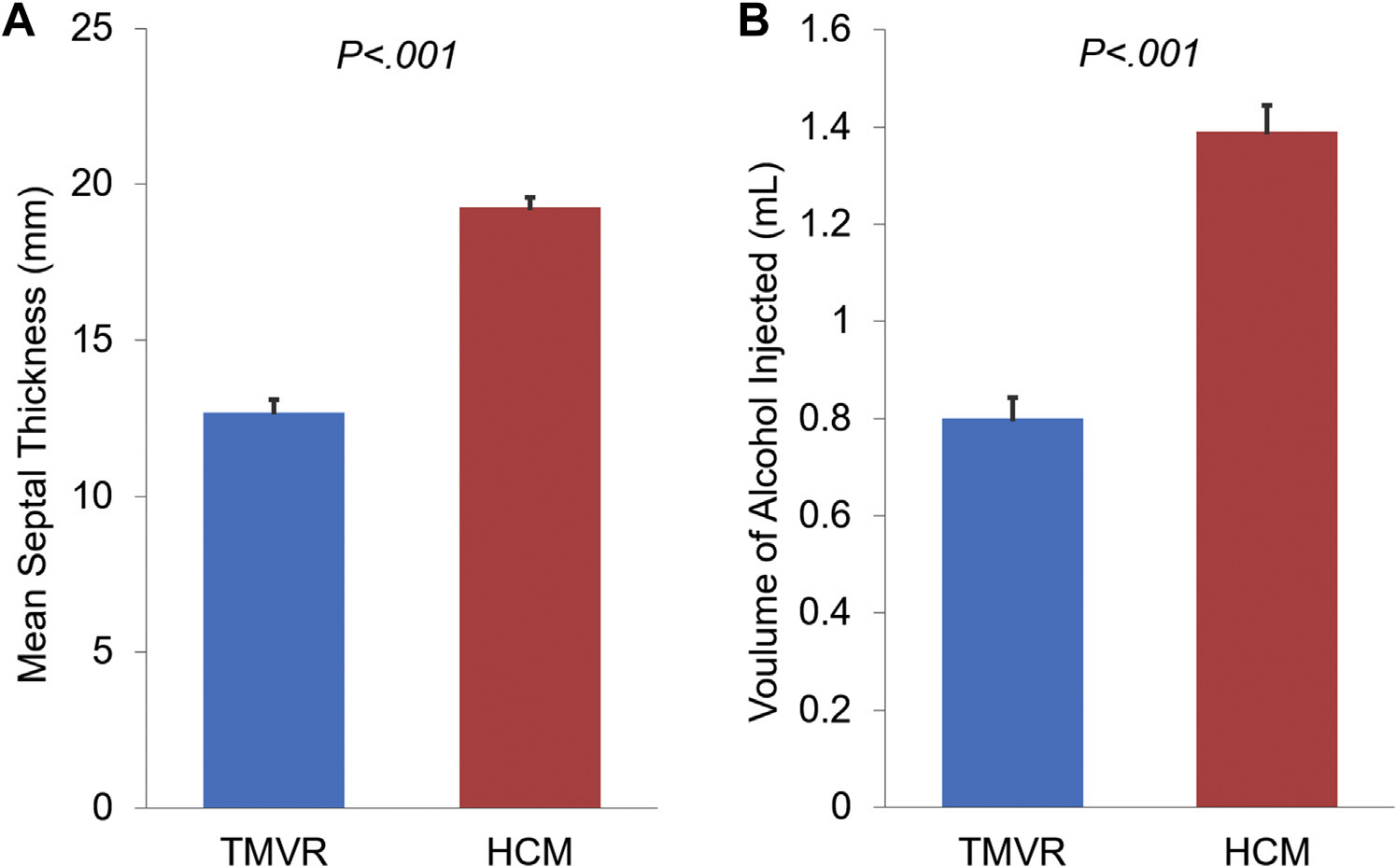
**Differences in mean septal thickness and volume of alcohol between the TMVR and HCM groups.** (**A**) The mean septal thickness (in millimeters) was measured on pre-ASA echocardiography and was higher in the HCM group (19.3 ​± ​3.1 ​mm) than in the TMVR group (12.7 ​± ​2 ​mm) ​(P ​< ​.001). (**B**) The mean volume of alcohol injected during the ASA procedure was also higher in the HCM group (1.4 ​± ​0.49 ​mL) than in the TMVR group (0.8 ​± ​0.2 ​mL) ​(P ​< ​.001). ASA, alcohol septal ablation; HCM, hypertrophic obstructive cardiomyopathy; TMVR, transcatheter mitral valve replacement.

A total of 102 patients were included in the study, with 80 patients in the HCM group and 22 patients in the TMVR group. Baseline characteristics are summarized in Table 1. The age and sex of patients in the TMVR group were similar to those in the HCM group (74 ​± ​10 ​years vs 73 ​± ​11 ​years; *P* ​= ​.613; 80% female vs 68% female; *P* ​= ​.24). Most comorbidities were more common in the TMVR group, except for chronic obstructive pulmonary disease, which was similar among both the groups (14% in the HCM group vs 18% in the TMVR group; *P* ​= ​.83). Coronary artery disease (59% vs 30%; *P* ​= ​.012), atrial fibrillation (50% vs 28%; *P* ​= ​.046), diabetes mellitus (46% vs 19%, *P* ​= ​.01), and chronic kidney disease (45% vs 25%, *P* ​= ​.062) were more common in the TMVR group than in the HCM group. The TMVR group had a higher rate of previous stroke or transient ischemic attack than the HCM group (36% vs 11%; *P* ​= ​.005). Patients in the TMVR group had a higher incidence of previous chest radiotherapy than that among patients in the HCM group (27% vs 1%; *P* ​< ​.001*)*. Two (9%) patients in the TMVR group and 13 (16%) patients in the HCM group had a preexisting permanent pacemaker (PPM) or defibrillator prior to the ASA procedure. The incidence of baseline conduction disease on pre-ASA electrocardiograms was similar in both groups (Table 1). Pre-ASA transthoracic echocardiography demonstrated increased interventricular septal wall thickness (19.3 ​± ​3.1 ​mm vs 12.7 ​± ​2 ​mm; *P* ​< ​.001), posterior wall thickness (12.4 ​± ​2.5 ​mm vs 10.8 ​± ​2.2 ​mm; *P* ​= ​.011), and left ventricular mass index (128.4 ​± ​32.1 ​g/m^2^ vs 102.2 ​± ​45.1 ​g/m^2^; *P* ​= ​.004) in the HCM group compared with those in the TMVR group (Central Illustration, Figure 2, and Table 1). ​The minimum septal thickness in the TMVR group was 10 ​mm.

**Table 1:** Baseline characteristics.

Characteristic	HCM group n = 80	TMVR group n = 22	*P* value^a^
Age, y	73 ​± ​11	74 ​± ​10	.613
Female sex	64 (80)	15 (68)	.24
Body mass index, kg/m^2^	31.7 ​± ​8	30 ​± ​7	.322
Atrial fibrillation	22 (28)	11 (50)	.046
Prior stroke/transient ischemic attack	9 (11)	8 (36)	.005
Hypertension	62 (78)	17 (77)	.982
Type 2 diabetes	15 (19)	10 (46)	.01
Coronary artery disease	24 (30)	13 (59)	.012
Chronic kidney disease	20 (25)	10 (45)	.062
Permanent pacemaker	13 (16)	2 (9)	.401
Chest radiation	1 (1)	6 (27)	<.001
Chronic obstructive pulmonary disease	13 (16)	4 (18)	.83
Pre-ASA electrocardiogram​^b^			
First-degree heart block	19 (28)	6 (30)	.887
Right bundle branch block	6 (9)	3 (15)	.436
Left bundle branch block	5 (7)	1 (5)	.703
Pre-ASA echo characteristics			
Ejection fraction, %	70.4 ​± ​5	67.2 ​± ​5.6	.013
Septal thickness, mm	19.3 ​± ​3.1	12.7 ​± ​2	<.001
Posterior wall thickness, mm	12.4 ​± ​2.5	10.8 ​± ​2.2	.011
Left ventricular mass index, g/m^2^	128.4 ​± ​32.1	102.2 ​± ​45.1	.004

Values are mean ​± ​SD or n (%).

ASA, alcohol septal ablation; HCM, hypertrophic obstructive cardiomyopathy; TMVR; transcatheter mitral valve replacement.

a Two-sample unpaired *t* test for continuous variables, χ^2^ test for categorical variables.

b Excludes patients with a PPM.

### TMVR group clinical characteristics

All patients in the TMVR group had New York Heart Association class III dyspnea on exertion because of mitral valve disease secondary to MAC. Eighteen percent of the patients had undergone cardiac surgery, and 6 patients had previously undergone a transcatheter aortic valve replacement. Echocardiography findings demonstrated that most (90%) patients had severe mitral stenosis, and 2 patients had combined severe mitral stenosis and severe mitral regurgitation. One patient had severe mitral regurgitation with mild mitral stenosis. The mean mitral valve gradient and mitral valve area were 9.2 ​± ​3.7 ​mm Hg and 1.49 ​± ​0.6 ​cm^2^, respectively. The mean left ventricular ejection fraction was 67.2% ​± ​5.6%. Concomitant valve disease was common in the TMVR group but was mostly mild to moderate. Twenty percent of patients had aortic stenosis (15% moderate and 5% severe). Twenty-three percent of patients had aortic regurgitation (14% moderate and 9% mild). Fifty percent of patients had concomitant tricuspid regurgitation (27% moderate and 23% mild). Tables 2 and 3 summarize the clinical characteristics of the patients in the TMVR group.

**Table 2:** Clinical characteristics and baseline echocardiographic data of the transcatheter mitral valve replacement group.

Characteristic	TMVR Group n = 22		
NYHA class III	22 (100)		
Previous cardiac surgery	4 (18)^a^		
Left ventricular ejection fraction, %	67.2 ​± ​5.6		
Mean MVG, mm Hg	9.2 ​± ​3.7		
Mitral valve area, cm^2^	1.49 ​± ​0.6		
Mitral valve disease^b^	Mild	Moderate	Severe
Mitral stenosis	1 (4.5)	1 (5)	20 (91)
Mitral regurgitation	10 (46)	5 (23)	3 (14)
Concomitant valve disease	Mild	Moderate	Severe
Aortic stenosis	0	3 (14)	2 (9)
Aortic regurgitation	2 (9)	3 (14)	0
Tricuspid regurgitation	5 (23)	6 (27)	0
Previous SAVR	4 (18)		
Previous TAVR	6 (27)		

Values are mean ​± ​SD or n (%).

MVG, mitral valve gradient; NYHA, New York Heart Association; SAVR, surgical aortic valve replacement; TAVR, transcatheter aortic valve replacement.

a Two patients had coronary artery bypass graft and SAVR and 2 patients had only SAVR.

b Two patients had combined severe mitral stenosis and severe mitral regurgitation.

**Table 3:** Clinical and echocardiographic characteristics of the transcatheter mitral valve replacement group.

Patient number	Age (y)	Sex	Prior SAVR/TAVR	Prior CABG	Concomitant valve disease	NYHA class	EF (%)	Mean MVG (mm Hg)	MV Area (cm^2^)
1	76	F	No	No	Mild-moderate TR	III	65	8	1.18
2	75	F	TAVR	No	Moderate TR	III	71	10	1.3
3	65	M	No	No	Mild-moderate AR, mild TR	III	75	8	1.3
4	93	M	TAVR	No	Mild periprosthetic AR	III	67	10	1.11
5	78	F	No	No	Mild AR	III	62	13	2.7
6	75	F	TAVR	No	Prosthetic AV	III	66	10	1.77
7	85	F	No	No	Moderate AR	III	75	7	1.3
8	69	F	No	No	Mild TR	III	65	6	1.2
9	63	F	No	No	Mild-moderate AS	III	70	7	1.06
10	84	F	No	No		III	65	6	1.34
11	81	M	SAVR	Yes	Prosthetic AV, previous MV repair	III	60	5	1.58
12	75	F	TAVR	No	Moderate severe AS, mild TR	III	80	6	0.9
13	82	F	No	No	Mild TR	III	70	10	1.04
14	68	F	SAVR	No	AV prosthesis/moderate TR	III	66	11	1.3
15	70	F	TAVR	No	Mild-moderate AR, Moderate AS	III	69	10	1.16
16	79	M	SAVR	No	Prosthetic AV, mild AR, mild-moderate TR	III	55	9	2.18
17	72	M	No	No		III	65	7	1.2
18	62	M	TAVR	No	Moderate AS	III	60	13	2.2
19	91	F	No	No	Moderate TR/AV papillary fibroelastoma/mild PR	III	65	8	2.4
20	70	M	SAVR	Yes	Bioprosthetic AV	III	65	3	2.8
21	60	F	No	No	Mild-moderate TR	III	72	20	0.8
22	53	F	No	No	Severe AS, Moderate MR	III	70	15	0.98

AR, aortic regurgitation; AS, aortic stenosis; AV, aortic valve; CABG, coronary artery bypass graft; EF, ejection fraction; F, female; M, male; NYHA, MR, mitral regurgitation; MV, mitral valve; MVG, mitral valve gradient; New York Heart Association; PR, pulmonic regurgitation; SAVR, surgical aortic valve; TAVR, transcatheter aortic valve replacement; TR, tricuspid regurgitation.

### Procedural characteristics

All patients in the TMVR group had only 1 branch of the septal perforator arteries injected. In the HCM group, most (84%) patients had 1 branch injected, whereas the remainder (16%) had 2 branches injected. The mean total volume of ethanol injected was higher in the HCM group than in the TMVR group (0.8 ​± ​0.2 ​mL in the TMVR group and 1.4 ​± ​0.49 ​mL in the HCM group; *P* ​< ​.001) (Figure 2). The CT characteristics before and after ASA in the TMVR group are summarized in Table 4. The median timing of cardiac CT after ASA was 49 ​days (IQR, 36-70 ​days). The average increase in LVOT area after ASA was 98 ​± ​88 ​mm^2^ in the TMVR group. The average frame neo-LVOT area (135 ​± ​89 ​mm^2^ vs 233 ​± ​111 ​mm^2^; *P* ​< ​.001) and skirt neo-LVOT area (275 ​± ​95 ​mm^2^ vs 357 ​± ​139 ​mm^2^; *P* ​= ​.005) also increased significantly after ASA. In patients with severe mitral regurgitation, the virtual valve frame to septum distance also increased after ASA (2.8 ​± ​2.9 ​mm vs 6.1 ​± ​3.2 ​mm; *P* ​< ​.001). Among the patients in the TMVR group, 6 patients did not show adequate improvement in the neo-LVOT after ASA and required further procedures. Two patients underwent radiofrequency ablation of the septum, 3 patients underwent the LAMPOON procedure, and 1 patient underwent both radiofrequency ablation and LAMPOON. There were no characteristics in our analysis that differentiated between patients who required further procedures after ASA and those that did not. Nineteen patients underwent successful transseptal TMVR. The median time from ASA to TMVR was 105 ​days (IQR, 56-210 ​days). Three patients did not undergo TMVR after ASA. One patient was deemed too high-risk for the TMVR procedure because of a small left ventricular cavity. Sudden cardiac death occurred in another patient before the planned TMVR procedure and 37 ​days after ASA. This patient had a new bifascicular block after ASA and was monitored in the hospital with telemetry for 4 ​days without any CHB noted, after which they were discharged from the hospital without ambulatory rhythm monitoring. They were seen as an outpatient in the clinic 7 ​days after the procedure and were doing well. The suspected cause of sudden death at 37 ​days in this patient was possible late CHB. The third patient decided not to undergo the TMVR procedure and died 10 ​months after ASA from heart failure.

**Table 4:** Computed tomographic characteristics before and after alcohol septal ablation.

Computed tomography measurements	Before ASA	After ASA	Change	*P* value
Virtual valve frame to septum distance (mm)	2.8 ​± ​2.9	6.1 ​± ​3.2	3.2 ​± ​2.6	<.001
LVOT area (mm^2^)	352 ​± ​79	456 ​± ​125	98 ​± ​88	<.001
Neo-LVOT area (mm^2^)	135 ​± ​89	233 ​± ​111	97 ​± ​60	<.001
Skirt neo-LVOT area (mm^2^)	275 ​± ​95	357 ​± ​139	96 ​± ​100	.005

Values are mean ​± ​SD.

ASA, alcohol septal ablation; LVOT, left ventricular outflow tract.

### Primary outcomes

**Figure. 3:**
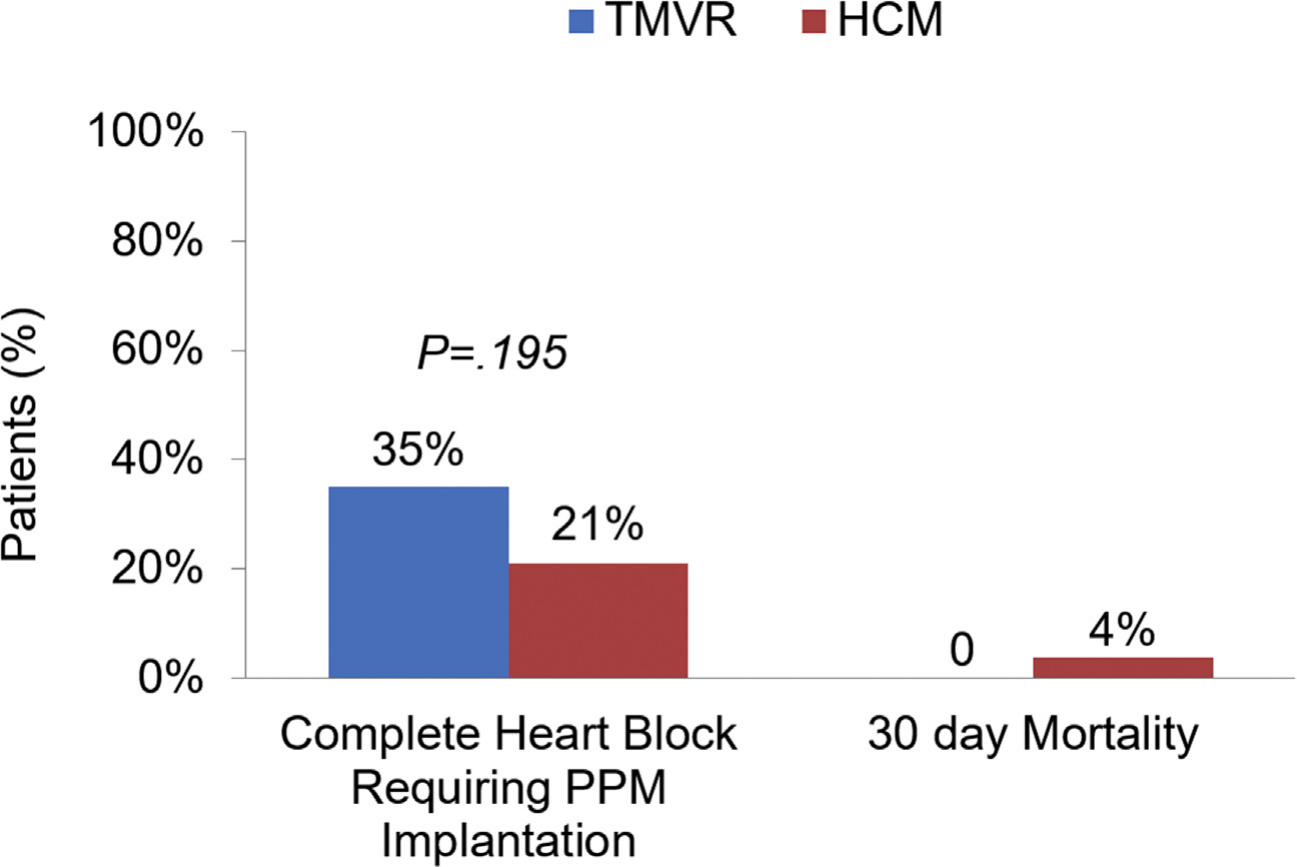
**Outcomes after alcohol septal ablation: Complete heart block and 30-day mortality.** The incidence of complete heart block requiring PPM after alcohol septal ablation was 35% in the TMVR group and 21% in the HCM group (*P* ​= ​.195). Thirty-day mortality was 0 in the TMVR group and 3.8% in the HCM group. HCM, hypertrophic obstructive cardiomyopathy; PPM, permanent pacemaker; TMVR, transcatheter mitral valve replacement.

**Figure. 4:**
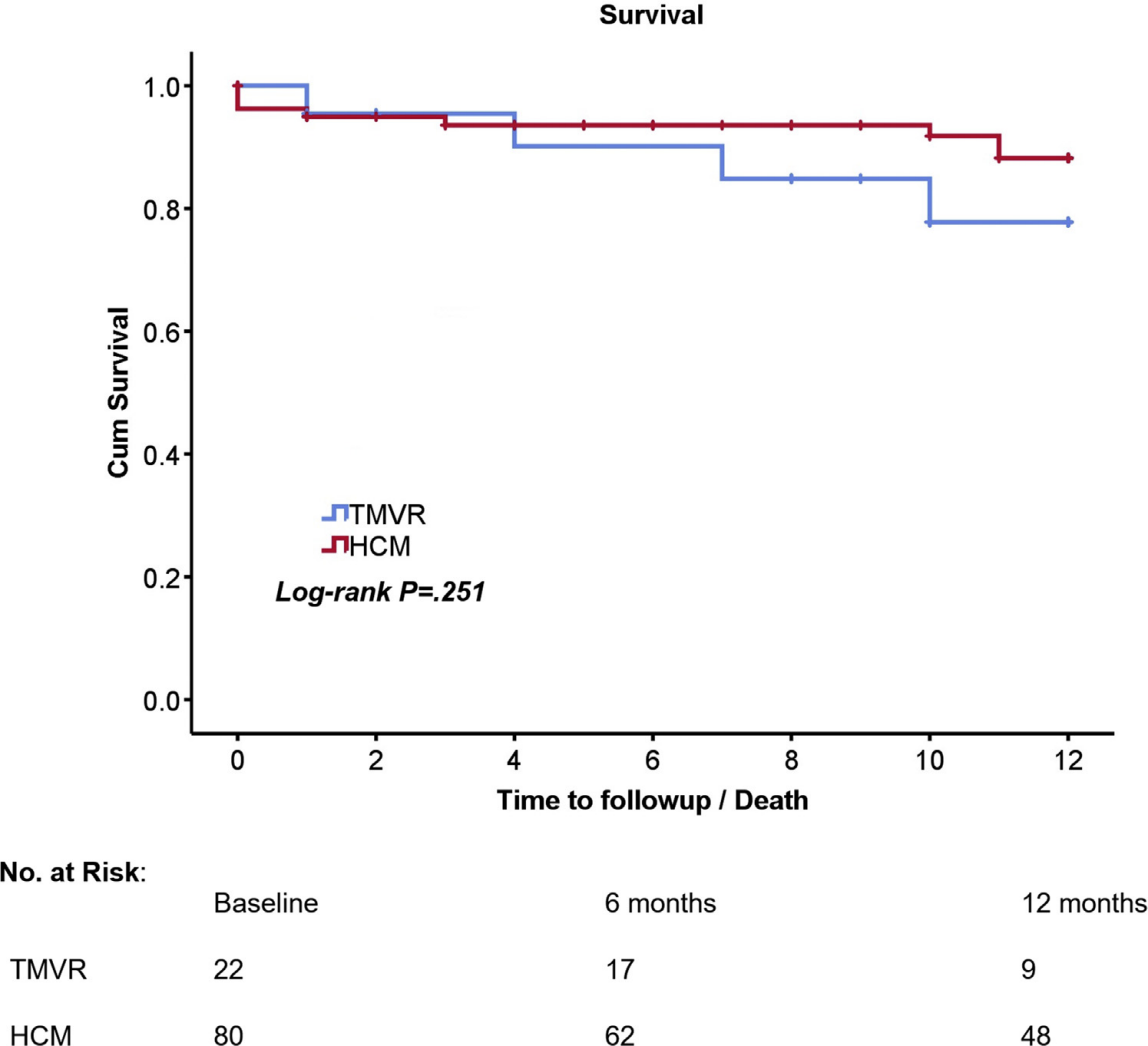
**Survival after alcohol septal ablation.** A Kaplan-Meier plot for the estimated 1-year survival after alcohol septal ablation for the TMVR and HCM groups. Cumulative survival was similar between the HCM and TMVR groups (*P* ​= ​.251). HCM, hypertrophic obstructive cardiomyopathy; TMVR, transcatheter mitral valve replacement.

The primary outcomes are summarized in Table 5. The incidence of CHB requiring PPM implantation at 30 ​days tended to be higher in the TMVR group than in the HCM group (35% vs 21%; *P ​=* ​.195) (Figure 3). Patients who had a PPM prior to the ASA procedure were excluded from the CHB analysis. No stroke or sustained ventricular tachycardia occurred in either group. There was no incidence of major bleeding in the TMVR group, and 3 patients in the HCM group experienced major bleeding, requiring intervention. In-hospital mortality was 0 for both the TMVR and HCM groups. Thirty-day mortality in the TMVR group was 0; however, 1 patient died at 37 ​days after ASA, presumably because of late CHB. Three patients in the HCM group died within 30 ​days of discharge after ASA. One patient died because of pulmonary embolism in the setting of malignancy, another patient died because of sepsis, and the cause of death was unknown for the third patient. The Kaplan-Meier survival analysis demonstrated a similar cumulative probability of survival to 1 ​year between the 2 groups (*P* ​= ​.251) (Figure 4).

**Table 5:** Alcohol septal ablation procedural outcomes.

Outcomes at 30 ​days	HCM group n ​= ​80	TMVR group n ​= ​22	*P* value^a^
Complete heart block	14 (21)	7 (35)	.195
Major bleeding	3 (4)	0	—
Sustained VT	0	0	—
Stroke	0	0	—
In-hospital mortality	0	0	—
30-Day mortality	3 (4)^b^	0^c^	—

Values are n (%).

HCM, hypertrophic obstructive cardiomyopathy; TMVR, transcatheter aortic valve replacement; VT, ventricular tachycardia.

a χ^2^ test conducted for the outcome of complete heart block.

b One patient died because of provoked pulmonary embolism in the setting of malignancy, another patient died because of sepsis, and the cause is unknown for the third patient.

c One patient died at 37 ​days after ASA from sudden cardiac death, presumably because of late complete heart block.

## Discussion

In this study, we demonstrated favorable safety with a preemptive ASA strategy with no instances of in-hospital or 30-day mortality. Treatments of patients with symptomatic mitral valve disease secondary to MAC are complex, and they are considered high-risk for cardiac surgery because of advanced age and comorbidities, thus representing an unmet clinical need for less invasive therapies.^9^ Our study is the largest single-center experience of ASA prior to TMVR for severe MAC and is the first, to our knowledge, to compare outcomes of ASA prior to TMVR to those of a control group of patients of HCM.

With the development of TMVR and the application of preemptive ASA to mitigate the risk of LVOT obstruction, more patients have the option to treat their severe mitral valve disease. However, the safety of ASA for septal reduction prior to TMVR has not been established. Therefore, in this study, we made several new observations: (1) preemptive ASA prior to TMVR was associated with a good safety profile with no intraprocedural or 30-day mortality; (2) rates of PPM implantation tended to be higher than those in an HCM control group, emphasizing the importance of careful monitoring after ASA; (3) patients undergoing TMVR for severe MAC had a significantly greater burden of comorbidities than an HCM control group; (4) septal thickness was considerably less in the TMVR group compared with that in patients with HCM, and corresponding lower doses of alcohol were used in this group; and (5) LVOT, frame neo-LVOT, and skirt neo-LVOT areas, measured on cardiac CT, increased significantly in the TMVR group after ASA.

Initial studies have demonstrated the feasibility of TMVR as a treatment strategy for patients with MAC.^2^ However, a significant limitation of TMVR is the risk of fatal LVOT obstruction. The threat of LVOT obstruction is also the most common exclusion criterion for patients undergoing screening for TMVR trials.^10^ Despite careful selection of patients, the rate of LVOT obstruction after deployment of the transcatheter valve is approximately 10% in patients with MAC. This complication is an independent predictor of mortality at 30 ​days and 1 ​year.^2^^,^^3^ Risk factors for LVOT obstruction include septal hypertrophy, acute aorto-mitral angle, and small left ventricular cavity.^11^^,^^12^ Strategies to mitigate the risk of LVOT obstruction after valve deployment are being evaluated. The LAMPOON procedure has been evaluated in a prospective trial of 30 patients and demonstrated feasibility.^13^ The limitations of this procedure include its technically challenging nature and the lack of feasibility when the anterior leaflet is severely calcified. ASA is a catheter-based procedure where alcohol is injected into the branch arteries supplying the septum, inducing a chemical infarct, leading to effacement of the septum and enlargement of the LVOT after several weeks. ASA is safe and effective in HCM and is indicated as part of the management of HCM in international guidelines.^14^ Urgent ASA has been used as a bail-out strategy for LVOT obstruction after deployment of transcatheter aortic valves and mitral valves.^15^, ^16^, ^17^ However, the remodeling to increase the LVOT after ASA requires several weeks to occur, and when LVOT obstruction is severe, mortality remains high.^18^ Preemptive ASA, performed 4 to 6 ​weeks before TMVR, is a strategy to increase the LVOT and allow time for remodeling. In the initial multicenter study investigating the feasibility of preemptive ASA in 30 patients undergoing TMVR who were at risk of LVOT obstruction, in-hospital and 30-day mortality was 6.7%.^5^ The incidence of CHB was 16.7%. In response to this publication, concerns were raised that the preemptive ASA technique may be limited by mortality and morbidity related to the risk of arrhythmias and pacing.^19^ Preemptive ASA before TMVR was used in the Mitral Implantation of Transcatheter Valves (MITRAL) ​trial, with a total of 7 patients (some of whom are included in this series) undergoing ASA, all of whom were alive and had successful TMVR at the 1-year follow-up.^2^ It is important to note that in our TMVR group, 6 patients required further procedures, either LAMPOON or radiofrequency ablation, with 1 requiring both the procedures. Radiofrequency ablation can be used to increase the neo-LVOT when ASA is unsuccessful or when coronary septal anatomy is unfavorable.^20^ Our analysis did not identify any unique or differentiating characteristics among patients who did not have a significant increase in their neo-LVOT after ASA. Further studies are required to refine patient selection and identify patients who would benefit most from either procedure or a combination of these procedures to reduce the risk of LVOT obstruction.

Additionally, upon comparing the characteristics of patients who underwent ASA for HCM, we noted that the TMVR group had a higher rate of comorbidities and, as expected, lesser degree of left ventricular hypertrophy and smaller maximal septal thickness. Despite these differences in anatomy and comorbidities, 30-day outcomes were similar between the 2 groups. Although there were no instances of 30-day mortality in this cohort of patients undergoing ASA prior to TMVR, 1 patient with a new bifascicular block experienced sudden death at 37 ​days after ASA, suggesting that they may have developed late CHB. This finding may warrant a more conservative pacing approach in patients who develop new bifascicular block after ASA prior to TMVR, with preemptive pacemaker implantation even in the absence of CHB in the early period after the procedure. Additionally, on the basis of our experience, we recommend 30-day ambulatory electrocardiogram monitoring in all patients undergoing ASA prior to TMVR who do not already have a PPM. This finding also prompted investigating survival outcomes beyond 30 ​days, and we established that the survival curves up to 1 ​year after ASA were similar between the 2 groups.

## Limitations

The study has several limitations, including the retrospective design with selection bias. Additionally, the study patients were from a single tertiary referral center with a large experience with the ASA procedure, which may limit the generalizability of our findings. The TMVR group size was small because of the relative novelty of TMVR and the stringent patient selection process for this procedure. Although patients with HCM are different from those with severe MAC being considered for TMVR, which results in confounding, left ventricular hypertrophy is consistently present in both the groups and is the underlying substrate for septal ablation. Additionally, a recent analysis of patients with HCM demonstrated that MAC is a common finding in patients with HCM,^21^ particularly in older and female patients, indicating important similarities between these 2 populations. Thus, comparing these 2 groups of patients provides new information regarding the acute procedural safety and outcomes of ASA in patients with severe MAC being considered for TMVR.

## Conclusions

Preemptive ASA, in preparation for TMVR in patients with severe MAC, is associated with safety and 30-day outcomes similar to ASA for obstructive HCM. Important differences in populations include a greater burden of comorbidities and a lesser degree of left ventricular hypertrophy in patients with severe MAC. Preemptive ASA before TMVR in patients with severe MAC is a safe and feasible strategy to mitigate the risk of left ventricular outflow obstruction. Careful monitoring for conduction changes after ASA with consideration for pacemaker implantation is required in this population.
